# Editorial: Acupuncture to treat pain in specific body regions

**DOI:** 10.3389/fpain.2024.1421548

**Published:** 2024-06-07

**Authors:** Carlo Maria Giovanardi, Alessandra Poini, Gianni Allais

**Affiliations:** ^1^ Association of Medical Acupuncturists of Bologna (A.M.A.B.), Research and Healthcare Institute, Bologna, Italy; ^2^ Women’s Headache Center and Service for Acupuncture in Obstetrics and Gynecology, University of Turin, Turin, Italy

**Keywords:** acupuncture, pain, Battlefield acupuncture (BFA), endometriosis, musculoskeletal pain, cancer survivors, acute pain analgesia, gouty arthritis pain

**Editorial on the Research Topic** Acupuncture to treat pain in specific body regions

“*Divinum est opus sedare dolorem*”. This ancient motto reflects how important it is for physicians to possess effective tools to counteract pain, one of the unfortunately necessary experiences for our body, which can accompany us several times throughout our lives. Undoubtedly, acupuncture, despite being a comprehensive medicine aimed at controlling all physiological functions of our organism and addressing any type of pathology that may manifest in the human body, has always been known in the West for its analgesic possibilities, to the extent that many individuals, including some within the medical community, consider it merely a pain-relief therapy. Certainly, for decades now, it has been demonstrated that acupuncture is effective in pain management through studies ranging from neurophysiological experiments in animal models to sophisticated neuroimaging techniques in humans, as well as numerous randomized and controlled trials re-evaluated through rigorous comparative meta-analyses.

One of the most fascinating characteristics of acupuncture in pain management is its ability to exert a ubiquitous analgesic action throughout the organism. The purpose of this issue of the journal is to gather articles that support the validity of acupuncture in pain management in vastly different parts of the body, with the aim of substantiating the aforementioned assertions.

Firstly, Choi et al., with a brilliant neurophysiological study titled “*What is the analgesic range of acupuncture stimulus for treating acupuncture pain?*”, demonstrate an extremely important general criterion in acupuncture pain treatment: the needle must always be correctly stimulated to achieve the desired analgesic effect, and it is advisable for it to be inserted near the locus of pain. In fact, the SEP amplitude values showed significant variations only when the needle at LI4 point was correctly stimulated after insertion.

In a condition such as acute gouty arthritis, a metabolic disorder characterized by recurrent and disabling painful episodes, the quality of life of affected patients can be severely compromised. Through a comprehensive systematic review of worldwide scientific literature in both English and Chinese, titled “*Electroacupuncture for acute gouty arthritis: a systematic review and meta-analysis of randomized controlled trials*,” Ni et al. demonstrate that electroacupuncture is superior to conventional medical therapies in pain control. Furthermore, the use of electroacupuncture also led to a significant reduction in serum uric acid levels.

Chiarle et al. address not only the problem of pain but also that of pain-related disability in patients suffering from a very serious condition, deep infiltrating endometriosis, in a paper titled “*Acupuncture for pain and pain-related disability in deep infiltrating endometriosis*.” These are all women who usually undergo repeated surgeries without ever completely eliminating the problem. In a trial that, for now, must be considered pilot, acupuncture demonstrates the ability to reduce dysmenorrhea (both in terms of number of days and intensity of pain), dyspareunia, and to a lesser extent, dyschezia, as well as the consumption of medications taken to control pain.

Fracchia et al., in their study “*Acupuncture in musculoskeletal pain: analysis of changes in pain perception using the NRS (Numeric Rating Scale)*”, analyze subjective changes in pain after acupuncture treatment in a large cohort of over 1,000 patients, with long-term monitoring spanning over 14 years. They found that acupuncture is a highly effective method for treating musculoskeletal pain. Furthermore, the improvement seems to be greater in certain areas of the body than in others.

Finally, Zhang et al. in a secondary analysis of a randomized controlled trial, “*Battlefield acupuncture for chronic musculoskeletal pain in cancer survivors: a novel care delivery model for oncology acupuncture*” brought out results with interesting clinical implications. Indeed, it appears that the clinical response to the first treatment with Battlefield acupuncture may be predictive of the overall response to treatment and that the rate of response to treatment may differ based on the location of the primary pain.

We are confident that this Research Topic will elicit a lot of interest among researchers, clinicians and patients. The works presented here provide interesting and innovative ideas in the field of acupuncture research and clinical practice.

We want to thank all the authors who contributed to this topic. Their work is helping improve the clinical management of patients with pain.

